# Templating Effect of Water-Soluble Anionic Phthalocyaninate on the Electropolymerization of 3,4-Ethylenedioxythiophene

**DOI:** 10.3390/polym15081854

**Published:** 2023-04-12

**Authors:** Oxana Gribkova, Varvara Kabanova, Alexey Yagodin, Aleksey Averin, Maria Teplonogova, Alexander Martynov, Alexander Nekrasov

**Affiliations:** 1 A.N. Frumkin Institute of Physical Chemistry and Electrochemistry of the Russian Academy of Sciences, 119071 Moscow, Russia; 2 Kurnakov Institute of General and Inorganic Chemistry of the Russian Academy of Sciences, 119071 Moscow, Russia

**Keywords:** PEDOT, electropolymerization, metal phthalocyaninates, spectroelectrochemistry, polyelectrolyte, atomic force microscopy, Raman spectroscopy

## Abstract

The electrochemical polymerization of 3,4-ethylenedioxythiophene (EDOT) was performed in the presence of a water-soluble anionic copper and zinc octa(3′,5′-dicarboxyphenoxy)phthalocyaninate containing 16 ionogenic carboxylate groups. The influences of the central metal atom in the phthalocyaninate and EDOT-to-carboxylate group ratio (1:2, 1:4, and 1:6) on the course of electropolymerization were studied using electrochemical methods. It has been shown that the polymerization of EDOT in the presence of phthalocyaninates proceeds at a higher rate compared to that in the presence of a low-molecular-weight electrolyte (sodium acetate). Studies of the electronic and chemical structure using UV–Vis–NIR and Raman spectroscopies showed that the use of copper phthalocyaninate leads to a higher content of the latter in PEDOT composite films. The 1:2 EDOT-to-carboxylate group ratio was found to be optimal for a higher content of phthalocyaninate in the composite film.

## 1. Introduction

Poly(3,4-ethylenedioxythiophene) (PEDOT) is one of the most popular conductive polymers, with a large number of applications [1]. The electrosynthesis of conductive polymers in the presence of large dopant anions improves the surface uniformity, mechanical properties, and thermal stability of the polymer films. The large anion is included in the composition of the film and is not removed from it during dedoping. In the case of PEDOT, the large doping anions such as polyelectrolytes (PE) with different structures [2], phthalocyaninate [3], polycatechol [4], poly(β-hydroxyethers) [5], etc., play the role of a doping anion and electrolyte and also serve as active components that affect the kinetics of electrosynthesis, as well as the structure and properties of the resulting polymer composites.

The preparation of hybrid materials based on conductive polymers and phthalocyanines is carried out using the following methods: (1) the electrocopolymerization of phthalocyanines molecules with electroactive monomers, for example, 3,4-ethylenedioxythiophene (EDOT) [6,7,8,9,10] or aniline [11]; (2) the use of phthalocyaninates with anionic substituents (carboxylic or sulfonic groups) as a charge-compensating anion in the electropolymerization of aniline [12,13], pyrrole [14,15], and EDOT [3]; (3) the chemical polymerization of EDOT in the presence of polystyrene sulfonic acid (PSSA) with copper phthalocyanine [16], poly(sodium styrene sulfonate), and transition-metal tetrasulfonated phthalocyanine as catalysts [17], and the chemical polymerization of aniline in the presence of hydrochloric acid and metal phthalocyaninates [18,19]. Another way to prepare hybrid material is the mixing of PEDOT–PSSA and copper phthalocyanine water dispersions. Layers were obtained by spin-coating the blended solutions [20,21]. The authors of [22] obtained bilayer structures. Aniline was electropolymerized on an electrodeposited copper phthalocyanine film.

The development of conductive composite films holding both PEDOT and metallophthalocyaninates is a promising way to obtain a multipurpose material for chemical sensors [3], biosensors [6,7], and thermoelectric [21] devices. It was shown in [20] that the use of mixtures of nickel phthalocyanine with PEDOT:PSSA in inverted perovskite solar cells as a hole-transporting layer led to enhanced photon absorption, with the improved film quality of perovskite increasing solar cell stability. The authors of [16] developed PEDOT:PSSA/CuPc layers polymerized on cloth fiber to electro-metalize stretchable copper conductive patterns. The perspective of electrochromic applications for the composites obtained by copolymerization of metal phthalocyanines and EDOT are presented in [7,9,10]. In [9], it was shown that electropolymerized Zn and/or Cu phthalocyanines holding EDOT residues had green and dark-gray colorations, and also absorptions in the NIR region, with high optical contrast ratios and very fast switching times. The presence of EDOT groups in phthalocyanines differentiates the colors of the composites’ oxidized forms [10].

To the best of our knowledge, electrochemical EDOT polymerization in the presence of metal phthalocyaninates (MPc) with ionic substituent groups has not been investigated. In our previous work [3], the use of water-soluble sodium salt of zinc phthalocyaninate containing carboxylate groups as doping anions in PEDOT electrochemical synthesis was carried out for the first time. We demonstrated the acceleration of EDOT electropolymerization in the presence of zinc phthalocyaninate at a ratio of EDOT-to-carboxylate groups of phthalocyaninate of 1:4 compared to that in the presence of a low-molecular electrolyte (sodium acetate). We discussed this acceleration in terms of the templating effect of the hydrophobic heterocyclic ring of phthalocyaninate with locally-ordered carboxylate groups in analogy with the EDOT template electropolymerization in the presence of polyelectrolytes. A similar acceleration of aniline electropolymerization in the presence of sulfonated nickel phthalocyaninate was observed in [12,13].

In this paper, we investigated the influence of the central metal ion in phthalocyaninates and ratios of EDOT-to-ionogenic groups of phthalocyaninates to reveal the nature of the templating effect on phthalocyaninates during EDOT electropolymerization.

## 2. Materials and Methods

### 2.1. Synthesis of Copper (Zinc) Octa(3,5-Pentoxycarbonylphenoxy)Phthalocyaninates

**Figure 1:**
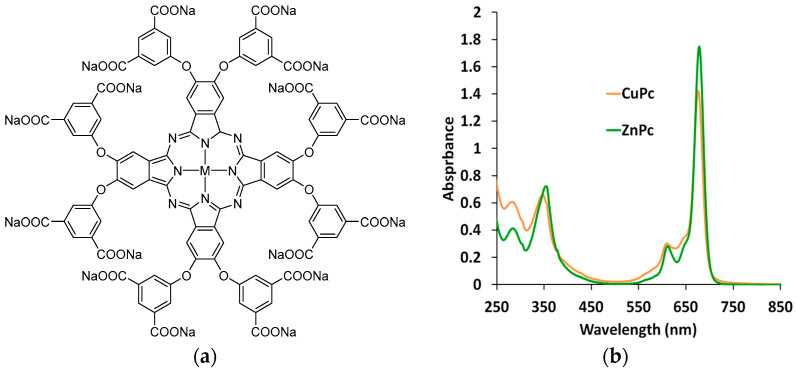
(**a**) Chemical structure of metal phthalocyaninates, where M = Cu and Zn, and (**b**) electron absorption spectra of metal phthalocyaninates aqueous solution (0.00125 M).

All organic reagents used for the synthesis were obtained from Sigma-Aldrich (St. Louis, MO, USA) and used without additional purification. Zinc octa(3,5-pentoxycarbonylphenoxy)phthalocyaninate (ZnPc) was synthesized by the cyclotetramerization of 4,5-[(3,5-bismethoxycarbonyl)phenoxy]phthalonitrile (see Figure S1 and its description), followed by the alkaline hydrolysis of ester groups, as previously reported [23]. Copper octa(3,5-pentoxycarbonylphenoxy)phthalocyaninate (CuPc) was synthesized using the analogous procedure: 4,5-[(3,5-bismethoxycarbonyl)phenoxy]phthalonitrile (320 mg; 0.58 mmol), CuCl (40 mg; 0.29 mmol), and DBU (130 µL; 0.87 mmol) was suspended in 7 mL of 1-pentanol and the mixture was brought to reflux under argon. The progress of the reaction was monitored by UV–Vis spectra. After 16 h, the resulting reaction mixture was evaporated. After that, a dark-blue sticky solid was sonicated with the mixture of water and 40 vol.% EtOH, the precipitate was filtered, washed with aqueous EtOH, washed off the filter with CHCl_3_ + 20 vol.% MeOH mixture, and the filtrate was evaporated. The target complex was isolated by column chromatography on SiO_2_ with a chloroform +2.5 vol.% methanol mixture. After evaporation of the chromatographic fractions, the copper octa(3,5-pentoxycarbonylphenoxy)phthalocyaninate was obtained as a dark-blue oily solid. Without additional characterization, this complex was dissolved in 5 mL of THF and added to the saturated solvent of NaOH in 50 mL of H_2_O:MeOH (1:5). The reaction was carried out at 40 °C under stirring with a magnetic stirrer for 2 h. The obtained precipitate was filtered, washed with chloroform and methanol, washed off with water, and evaporated. The target complex CuPc was obtained as a green solid (276 mg; overall yield 64%). UV–Vis (H_2_O), Figure 1b: λ_max_ (logε) 228 (4.68), 346 (4.72), 610 (4.38), 676 (5.06). 

### 2.2. EDOT Electropolymerization in the Presence of CuPc and ZnPc

Our earlier studies [3] showed that the films of PEDOT–ZnPc (1:4) composites obtained on bare FTO electrodes in potentiostatic (PS) and galvanostatic (GS) modes had bad surface uniformity and no film can be produced by potentiodynamic (PD) synthesis. In the case of PEDOT–MPc composite electrodeposition on a thin PEDOT sublayer, the films were homogeneous with good adhesion to the substrate. To improve the film formation and interfacial charge transfer performance between the polymer film and ITO electrode, the authors of [24] also used an electrodeposited PEDOT–PSSA sublayer. Therefore, in this work, all PEDOT–MPc composite films were electrodeposited onto a thin sublayer.

The following reagents were purchased from Sigma-Aldrich (St. Louis, MO, USA). EDOT was distilled under argon, and a freshly distilled product was used. The PEDOT sublayers were synthesized on FTO electrodes (Solaronix SA, Aubonne, Switzerland) in the GS (0.05 mA/cm^2^) mode in an aqueous solution containing 0.01 M EDOT and 0.02 M poly-(2-acrylamido-2-methyl-1-propanesulfonic acid) (PAMPSA) prepared by diluting its 15% aqueous solution (Mw ≈ 2,000,000). Electrodeposition of the sublayer was carried out until an electropolymerization charge of 7 mC/cm^2^ was reached. A platinum foil was used as a counter electrode, and a saturated silver–silver chloride electrode was used as a reference electrode. All potentials in this work are presented relative to this electrode.

EDOT electropolymerization in the presence of ZnPc and CuPc was carried out in 0.01 M EDOT aqueous solution at different ratios of EDOT-to-carboxylate groups of phthalocyaninate. We chose three ratios of 1:2 (0.01 M EDOT and 0.00125 M MPc), 1:4 (0.01 M EDOT and 0.0025 M MPc), and 1:6 (0.01 M EDOT and 0.0038 M MPc), which, according to the structure of phthalocyaninate (Figure 1a), provided the different number of the carboxylate groups necessary to compensate charges of the forming PEDOT chains. Phthalocyaninates were dissolved in water, then EDOT was added and intensively stirred for 1 h with heating to ~60 °C. Polymerization was carried out in a three-electrode cell in an aqueous medium on optically transparent electrodes consisting of a fluorine-doped tin oxide layer on a glass substrate (FTO electrodes) with an area of 1.6 cm^2^, either bare or covered with a PEDOT sublayer (the sublayer deposition procedure is given above). The electrodeposition of PEDOT–MPc composite films was carried out in PD mode in the potential range of −0.0 ÷ 0.9 V at a sweep rate of 50 mV/s, in PS mode at the potential of 0.85 V and GS mode at a current density of 0.15 mA/cm^2^. The charge spent for the synthesis of the composite films was 50 mC/cm^2^.

For comparison, EDOT polymerization was performed on FTO electrodes with a PEDOT–PAMPSA sublayer in the presence of a low-molecular-weight electrolyte, sodium acetate (NaOAc). Synthesis was carried out in aqueous solutions containing 0.01 M EDOT and 0.02 M NaOAc at the same conditions as the PEDOT–MPc films.

The electrochemical parameters during the electrosynthesis, electrochemical and spectroelectrochemical studies were controlled and recorded using an Autolab PGSTAT302N potentiostat (Metrohm, Utrecht, The Netherlands). Spectroelectrochemical studies of the obtained films at fixed potentials in an aqueous solution of 0.5 M NaClO_4_ (350–900 nm) were carried out using an Avantes 2048 (Avantes B.V., Apeldoorn, The Netherlands) CCD-detector single-beam spectrophotometer.

Electronic absorption spectra of the obtained composite films on FTO electrodes in the UV–Vis–NIR (up to 1850 nm) range were registered in air using a Shimadzu UV 3101 spectrophotometer (Shimadzu Europa GMBH, Duisburg, Germany).

Raman spectra of the films on FTO electrodes were recorded using a Renishaw InVia Raman confocal microscope (Renishaw Inc., West Dundee, IL, USA) with excitation by a 633 nm He–Ne laser with an intensity of less than 31 μW. The samples were mounted on a nonreflecting surface of the Raman microscope stage. The spectra were registered in the 100–2000 cm^−1^ range at the integration time of 100 s with a spectral resolution of ~3 cm^−1^ (1800 mm^−1^ grating) using a 50× objective (NA 0.5, FN 26.5), the irradiated spot diameter being ~2 μm.

The AFM images of the composite films were obtained using an atomic force microscope (AFM) Enviroscope with a Nanoscope V controller (Bruker, Billerica, MA, USA) in the tapping mode.

Scanning electron microscopy (SEM) images were obtained using a Tescan Amber GMH high-resolution electron microscope (Tescan Orsay Holding, a.s., Brno–Kohoutovice, Czech Republic) at an accelerating voltage of 1 kV. To prevent image artifacts, the samples were analyzed without the deposition of any conducting layer on their surface. Electron probe microanalysis was conducted using integrated energy dispersive spectroscopy (EDS) detector (Oxford Instruments Ultim Max 100 mm^2^, Abingdon, UK).

## 3. Results and Discussion

### 3.1. Spectroscopy of Metal Phthalocyaninates Aqueous Solutions

Figure 1 shows the chemical structure and electron absorption spectra of the phthalocyaninates aqueous solutions. The absorption bands in the UV–Vis spectra of phthalocyanines arise mainly from excitations between occupied and vacant molecular orbitals localized on the macrocyclic ligands [25]. The most intense band at 660–680 nm—the so-called Q-band—arises from HOMO–LUMO excitation, and is accompanied by a well-resolved low-intensity vibrational satellite at 630 nm.

Aggregation of the phthalocyanine complexes via stacking interactions leads to the appearance of the new band at 630–650 nm, which worsens the resolution of the vibrational satellite. Thus, the shape of the spectrum in this region is very sensitive to the state of the molecules in the solution. From Figure 1b, it is clear that the spectrum of CuPc absorbance in the range of 630–650 nm is higher than that in the spectrum of ZnPc, which testifies for a more aggregated state of the former phthalocyaninate in the solution [26].

The difference between the aggregation states of Cu(II) and Zn(II) Pcs results from the coordination properties of these metal centers in the macrocyclic environment. The zinc cation can coordinate solvent molecules (water in the present case) placed orthogonal to the macrocyclic plane, thus inhibiting intermolecular stacking interactions [27]. In contrast, the copper cation remains in the square-planar state, and thus its complex has a higher tendency to aggregate [28].

### 3.2. Electrochemical Synthesis of PEDOT–MPc Composites

**Figure 2:**
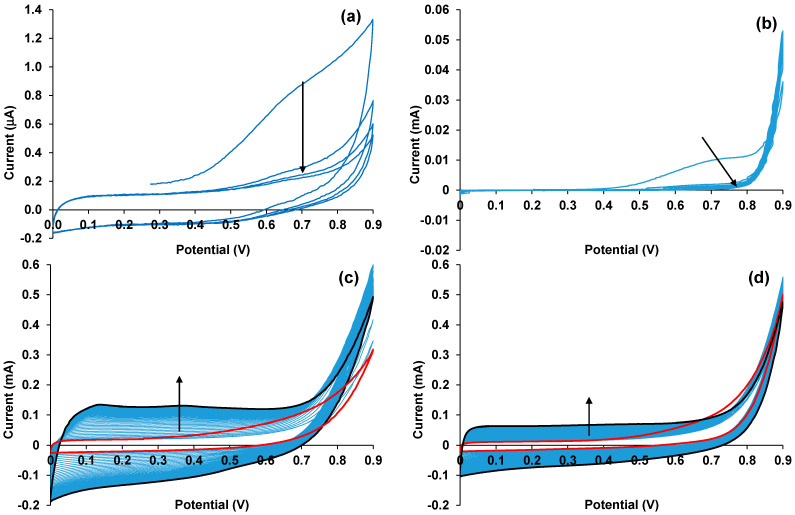
Cyclic voltammograms in aqueous solutions: (**a**) bare FTO electrode in 0.0005 M CuPc and 0.25 M NaClO_4_; (**b**) bare FTO electrode in 0.01 M EDOT and 0.00125 M CuPc; (**c**) FTO electrode with a PEDOT–PAMPSA sublayer in 0.01 M EDOT and 0.00125 M CuPc; and (**d**) FTO electrode with the sublayer in 0.01 M EDOT and 0.00125 M ZnPc. The potential scan rate was 50 mV/s. The red lines in (**c**,**d**) represent the first cycle, and the black lines, the 40th cycle.

The cyclic voltammograms of the electrooxidation of CuPc on the bare FTO electrode are presented in Figure 2a. One can see that in the first cycle, the phthalocyaninate is oxidized in the 0.4–0.9 V potential range and then the current gradually decreases as the products of phthalocyaninate oxidation block the electrode surface. Figure 2b shows that during the electrooxidation of EDOT–CuPc, a 1:2 mixture on the bare FTO electrode CuPc is oxidized at lower potentials than those needed for EDOT oxidation (>0.85 V), and also blocks the FTO surface. As a result, no polymer film grows on the electrode. Expansion of the potential scanning range to an anodic region did not improve the situation. We observed a similar effect for EDOT–ZnPc 1:4 mixture electrooxidation on a bare FTO electrode in [3]. 

Therefore, in the next stage, we used a similar approach as in [3], i.e., electrosynthesis on FTO electrodes covered with the PEDOT–PAMPSA sublayer. One can see from Figure 2c that the character of the CV shape evolution differs cardinally from the electropolymerization PEDOT–CuPc 1:2 mixture on the bare FTO electrode, showing no CuPc oxidation current in the 0.4–0.9 V range, and the EDOT oxidation currents are higher by an order of magnitude. The CV curves are similar to those registered during EDOT electropolymerization in an aqueous medium in the presence of flexible-chain polyacids [29].

Similar but slightly lower currents were observed during PD synthesis in the ZnPc solution (Figure 2d), indicating a lower polymerization rate in this case. In the first cycles of the electrosynthesis in PD mode, we did not observe any differences in the EDOT oxidation potentials depending on the nature of the central atom of the phthalocyaninates. Therefore, at this point, we cannot suppose some catalytic effect of copper on the synthesis. 

**Figure 3:**
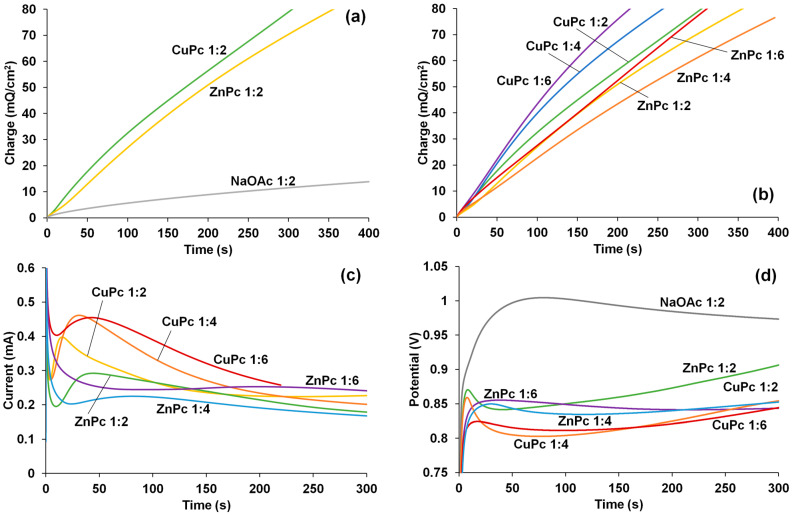
Time dependences of charge (**a**,**b**) and current (**c**) obtained during PS synthesis, and potential (**d**) obtained during GS synthesis in the presence of MPc.

Figure 3a shows the time dependences of the charge of EDOT–MPc polymerization in PS mode. It is apparent that the rate of synthesis is significantly higher in MPc solutions compared to NaOAc solutions at the ratio of 1:2.

This acceleration may be explained by the templating effect of phthalocyaninate molecules, which consists of the preliminary association of EDOT molecules with the hydrophobic macroheterocyclic ring of the phthalocyaninates. In addition, the high local concentration of the carboxylate groups facilitates the PEDOT chain growth due to a more efficient compensation of the positive charges of the formed radical cations.

If we compare the dependences of charge at different ratios for ZnPc and CuPc (Figure 3b), one can see that the synthesis rate in CuPc solutions decreases in the order 1:6 > 1:4 > 1:2. This sequence may be explained by the incorporation of higher quantities (see the below discussion of UV–Vis–NIR and Raman spectra) of large CuPc molecules, which hinder interchain electron transport in PEDOT films. Corresponding dependences for EDOT polymerization in ZnPc solutions are close, but the synthesis in EDOT–ZnPc 1:4 proceeds a bit slower.

The current transients obtained during EDOT polymerization in the presence of MPc with different ratios presented in Figure 3c support the above supposition. In the case of ZnPc 1:2 and all CuPc solutions, the current increases in the first 10–40 s of the synthesis and then slowly decreases. For ZnPc with 1:4 and 1:6 ratios, the currents decrease in the early stages and then change insignificantly. The intense decrease in current for EDOT polymerization in the presence of CuPc may be connected both with the diffusion control and growth of the film resistance due to its increasing content of CuPc. An indirect proof for domination of the later factor is flatter current transients in the case of ZnPc, whose content in the film, according to the Raman spectra presented below, is significantly lower. However, at this time, we have no method allowing us to quantitatively discriminate between the influence of these two factors.

During GS synthesis, EDOT polymerization in NaOAc proceeds at a high potential of about 1 V (Figure 3d). In the cases of ZnPc 1:2, CuPc 1:2, and CuPc 1:4, we first observe rapid growth, then a fall, and again, an increase in potential. In the characterization of time dependences of potential for ZnPc 1:4 and 1:6, CuPc 1:6 is more typical for PEDOT synthesis in aqueous solutions with inorganic electrolytes and polyelectrolytes [30]. 

Thus, the most significant acceleration of EDOT electropolymerization in the presence of ZnPc is observed at the lowest concentration ratio 1:2. The EDOT polymerization in CuPc-containing solution at all ratios proceeds at a higher rate compared to any of the syntheses in the presence of ZnPc. 

### 3.3. Electron Spectroscopy in the UV–Visible and Near-IR Regions

**Figure 4:**
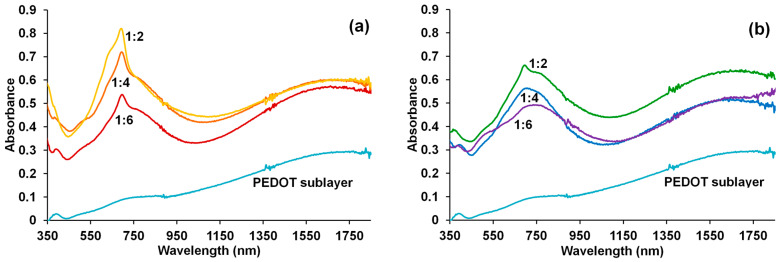
Electronic absorption spectra of PEDOT–CuPC (**a**) and PEDOT–ZnPC (**b**) composite films on FTO electrodes obtained in PS mode (charge spent for the syntheses is 50 mC/cm^2^) in solutions with different EDOT-to-carboxylate ratios. The spectrum for the PEDOT sublayer is given for comparison.

The electron absorption spectra in the UV–Vis–NIR region of the obtained PEDOT composite films are presented in Figure 4. For all films, we observed a shoulder of broad absorption maximum near 680–780 nm and an absorption plateau near 1640 nm, which are characteristic of PEDOT in the polaron state [31], as compared with the PEDOT–PAMPSA sublayer whose spectrum corresponds to the highly conductive bipolaron state of PEDOT [31,32,33].

One can see a distinct maximum at 696 nm in PEDOT–CuPc films (Figure 4a). In the case of PEDOT–ZnPc composites at the ratios of 1:4 and 1:6, only a small plateau near 700 nm is observed, while at ratio 1:2, there is a pronounced maximum at 696 nm (Figure 4b). This absorption corresponds to the monomer form of phthalocyaninate (Figure 1b). Interestingly, both PEDOT composites synthesized in the same conditions and with the same polymerization charge have a different level of absorption (supposedly thickness) depending on the ratio of the components. We see the highest absorption for a 1:2 ratio in both PEDOT–MPc composites. The highest maximum of phthalocyaninate is observed in the PEDOT–CuPc film, which has the maximum synthesis rate. We assume that in the case of higher MPc contents in the synthesis solution, an excess of bulky MPc molecules may hinder the precipitation of the oxidized oligomers on the electrode, preventing the formation of a compact composite layer. Moreover, in the case of the PEDOT–CuPc film, a more pronounced shoulder is observed in the 635 nm region, which, according to Figure 1b, may correspond to the aggregates of phthalocyaninates.

### 3.4. Raman Spectroscopy

**Figure 5:**
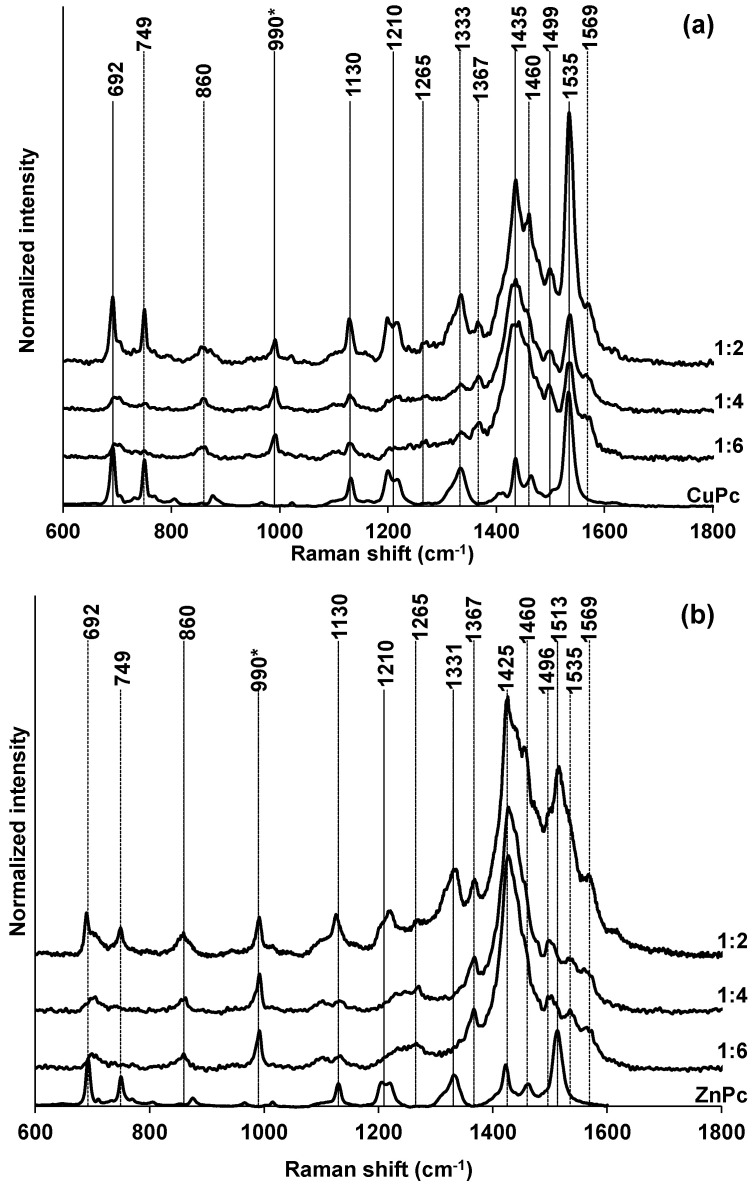
Normalized (PEDOT line near 990 cm^−1^) Raman spectra of PEDOT–CuPc (**a**) and PEDOT–ZnPc (**b**) composite films on FTO electrodes obtained in PS mode (charge spent for the synthesis: 50 mC/cm^2^) in solutions with different EDOT-to-carboxylate ratios. Spectra of phthalocyaninates are normalized by arbitrary values for easier visual comparison.

Figure 5 shows normalized Raman spectra of the composite films on FTO electrodes. The normalization was carried out at the intensity of PEDOT vibration near 990 cm^−1^, which is ascribed to the bending of the oxyethylene ring [2]. Other PEDOT vibrations are obviously presented in the spectra (the line positions according to [2] are put in parentheses): 1569(1558) cm^−1^—asymmetric C=C stretching in the bipolaron structure [34]; 1496(1502) cm^−1^—asymmetrical stretching of C=C; 1425(1430) cm^−1^—symmetrical stretching of C=C; 1367(1371) cm^−1^—stretching of the C_β_–C_β_ bonds in the thiophene ring; 1265(1260) cm^−1^—stretching of the C_α_–C_α′_ bonds between thiophene rings in the polymer chain; and 860 cm^−1^ (similar vibration was observed near 850 cm^−1^ in [33] for the oxidized state of PEDOT but remained unattributed). Minor deviations in the vibration frequencies are likely connected with a slightly different oxidation state of PEDOT in the composites with phthalocyaninates.

Characteristic Raman vibrations of the MPc are observed near, 692(692 [35]) cm^−1^—the macrocycle deformation; 749(754 [35]) cm^−1^—C–H and C–C–C deformation; 1130(1114 [35]) cm^−1^—C–C stretching and C–H deformation; 1210 (1220 [36]) cm^−1^—C_γ_–H in-plane bending in indole; 1331(1325 [35]) cm^−1^—pyrrole ring deformation, and 1460(1438 [35]) cm^−1^—isoindole ring stretching. Frequencies of the vibrations near 1520 cm^−1^ (C–N–C stretching in the isoindole ring [36]) depend on the central metal atom: 1535(1530) cm^−1^ for CuPc and 1515(1505) cm^−1^ for ZnPc.

The comparison of the intensities of the most representative Raman vibrations of MPc near 1520 cm^−1^ in the spectra of composite films confirms the conclusion formed by UV–Vis spectroscopy: the highest content of phthalocyaninate is achieved by performing electropolymerization in solutions of a 1:2 EDOT-to-carboxylate ratio. The use of CuPc leads to a much higher content of the phthalocyaninate in the composite.

### 3.5. Cyclic Voltammetry

**Figure 6:**
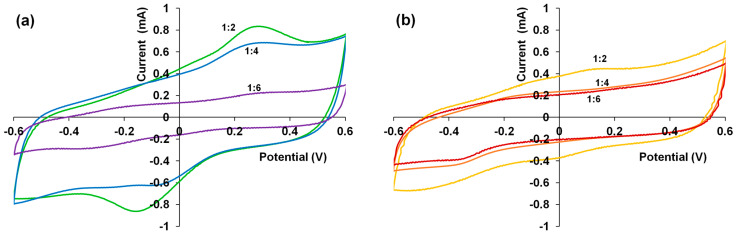
Cyclic voltammograms of PEDOT composite films synthesized in the presence of CuPc (**a**) and ZnPc (**b**), recorded in 0.5 M NaClO_4_ aqueous solution at a potential scan rate of 50 mV/s.

Before spectroelectrochemical studies, the films were subjected to 5 CV cycles (50 mV/s) in 0.5 M NaClO_4_ solution in the potential range from –0.6 to 0.6 V. The final CV curves are presented in Figure 6. Despite the equal electrodeposition charge, the composite films demonstrate different redox charges. The CVs of PEDOT–CuPc and PEDOT–ZnPc 1:2 films have pronounced anodic current maxima near 0.2–0.3 V and cathodic current near −0.15 V. The CVs of PEDOT–ZnPc (1:4 and 1:6) films looked like the CV of ordinary PEDOT film prepared in an aqueous solution [32].

It is clearly seen that the redox charges (Figure 6a), as well as the optical absorbance (Figure 4a), are higher for 1:2 and 1:4 ratios compared to a 1:6 ratio for PEDOT–CuPc films, which also corresponds to a higher content of CuPc in these films, as observed by Raman spectroscopy (Figure 5a).

One can explain these phenomena as follows. CuPc is more aggregated in solution than ZnPc (Figure 1b) and CuPc aggregate can associate with more EDOT monomers nearby, thus ensuring their higher local concentration compared to weakly-aggregated ZnPc. This contributes to both more rapid EDOT polymerization and a higher content of CuPc in the composite film. Moreover, CuPc remains aggregated inside the composite film, which is supported by the presence of the 630 nm absorption in the spectra of dry films (Figure 4a).

### 3.6. Spectroelectrochemical Studies

**Figure 7:**
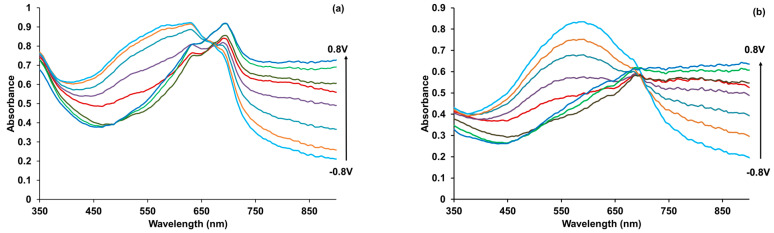

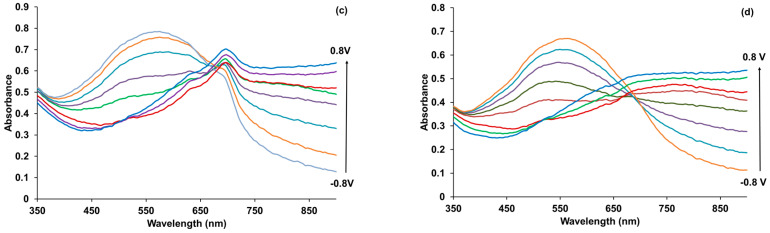
Optical absorption spectra of PEDOT films prepared in CuPc 1:2 (**a**), ZnPc 1:2 (**b**), CuPc 1:4 (**c**), and ZnPc 1:4 (**d**), measured at different potentials (step 0.2 V) in a 0.5 M aqueous solution of NaClO_4_.

The absorption spectra taken at fixed potentials for the PEDOT composite films at low potentials show a band near 570 nm caused by π–π*-transitions in the PEDOT reduced form (Figure 7). With increasing potential (oxidation), the π–π* band intensity decreased, and simultaneously, absorption in the longer region at 750 nm increased (the polaron form of oxidized PEDOT). Further, upon oxidation of the films, the maximum phthalocyaninate near 690 nm becomes more and more well-pronounced. This is observed at all potentials for PEDOT–CuPc composites at all ratios and for ZnPc 1:2. In the case of PEDOT–ZnPc with 1:4 and 1:6 ratios, a shoulder near 690 nm is almost invisible. The spectra of PEDOT–CuPc at all ratios and PEDOT–ZnPc 1:2 at higher potentials are also characterized by the appearance of an additional maximum or shoulder near 630 nm. In accordance with Figure 1b, this absorption may be ascribed to the phthalocyaninate aggregation. For CuPc 1:4, the aggregation becomes less clearly pronounced.

### 3.7. The Morphology of PEDOT–MPc Composites

**Figure 8:**
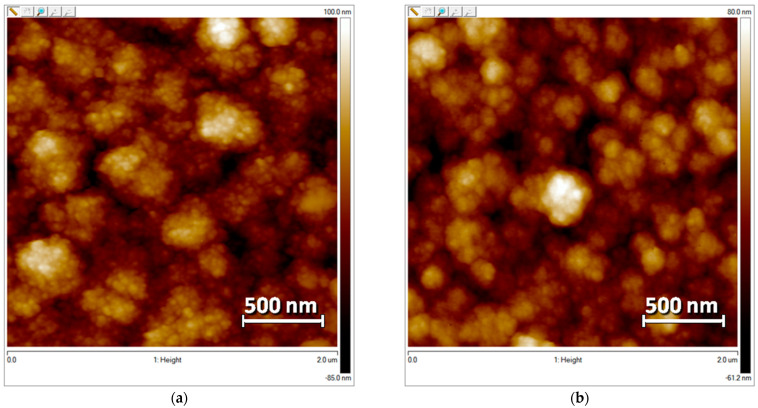

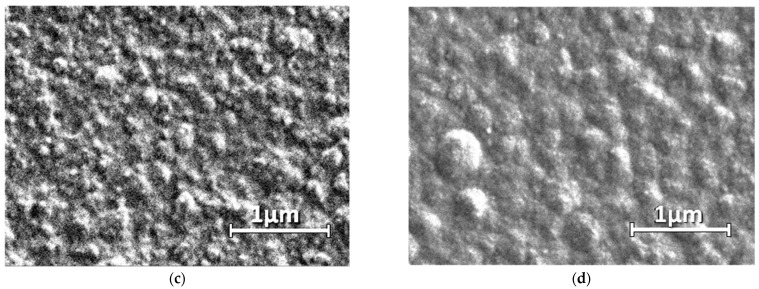
AFM (**a**,**b**) and SEM (**c**,**d**) images of the surfaces of PEDOT–CuPc 1:2 (**a**,**c**) and PEDOT–ZnPc 1:2 (**b**,**d**) films on FTO electrodes.

The composite films were characterized using AFM (Figure 8a,b) and SEM (Figure 8c,d, and Figure S2) methods. PEDOT–CuPc and PEDOT–ZnPc composites have very similar globular morphology with close roughness. The surface of the films consists of isolated globules with a lateral size of 150–350 nm and a height of 40–80 nm. PEDOT–CuPc 1:2 demonstrates the highest roughness (Rq = 25.3 nm) compared to other composite films.

The chemical composition of the films was tested using EDS. Unfortunately, their thickness was too low to obtain reliable quantitative data on the metal-to-sulfur ratio. Nevertheless, in all of the EDS spectra, the weak characteristic lines of metal atoms were present (Figure S3 for CuPc) supporting the incorporation of phthalocyanines into the polymer matrix.

## 4. Conclusions

As a result of this study, we have developed an electrochemical procedure for creating new composite material, which combines good film-forming and adhesion properties of the conducting polymer PEDOT and spectral characteristics of metal phthalocyaninates (MPc). It was found that composite PEDOT–MPc films cannot be electrodeposited onto bare FTO electrodes because of the blocking of the electrode surface by the products of MPc oxidation, which occur at the potentials lower than those necessary for EDOT electropolymerization. Fortunately, the use of a thin PEDOT–PAMPSA sublayer pre-electrodeposited onto the FTO electrodes prevented MPc oxidation, which is obviously an undesirable process, especially if one intends to use MPc as a complex counter anion in EDOT polymerization. 

Experiments on the electrodeposition of both PEDOT–CuPc and PEDOT–ZnPc films on the sublayer in the potential cycling regime did not reveal any changes in the starting potential of EDOT oxidation. Therefore, at this time, we do not have evidence of the catalytic effect of the central metal atom in the MPc. At the same time, further experiments in potentiostatic and galvanostatic regimes showed, respectively, in general, higher currents (initial stage) and lower potentials for the PEDOT–CuPc electrosynthesis compared to those of PEDOT–ZnPc. However, as the electrodeposition proceeded in a potentiostatic regime, the currents in the case of PEDOT–CuPc gradually decreased down to the values typical for PEDOT–ZnPc electrodeposition. This may be explained by a competition between the templating effect of PEDOT–CuPc accelerating the process, and a more rapid increase in the film resistance decelerating the process as the growing number of large macroheterocyclic molecules enter the film. The latter fact may hinder interchain electron transfer in the bulk of the film. At the same time, we clearly understand that additional experiments (for example, in situ Raman spectroelectrochemistry) are needed to prove these suppositions.

The higher content of CuPc in the composite films compared to ZnPc was further confirmed by UV–Vis–NIR and Raman Spectroscopy, the largest MPc content being observed in the films prepared in the solutions with a 1:2 EDOT-to-carboxylate ratio.

Electrochemical (cyclic voltammetry) studies of the obtained composite films revealed the appearance of the new redox pair (near 0.2–0.3 V in anodic and −0.2–0.0 V in the cathodic region), which can be attributed neither to PEDOT nor phthalocyaninate. Spectroelectrochemical studies in these ranges of potentials did not clarify the nature of this redox pair, revealing no spectral changes associated with the Q-band of the MPc. Further Raman spectroelectrochemistry experiments may help elucidate the nature of this redox pair.

AFM and SEM studies of the morphology of obtained composite films did not reveal a strong dependence of the average roughness or thin structure of the surface objects on the nature of the MPc central atom or EDOT-to-carboxylate ratio. However, the PEDOT–CuPc films may be considered slightly rougher. 

Considering possible applications of the obtained composite films, it is also useful that the composite layers can be electrodeposited only onto the PEDOT sublayer, which usually serves as a hole-transporting and electron-blocking layer in various devices of organic electronics. Further electrophysical studies will be carried out to estimate the applicability of this material in these applications.

## Data Availability

Not applicable.

## Supplementary Materials

The following supporting information can be downloaded at: https://www.mdpi.com/article/10.3390/polym15081854/s1, Figure S1. Synthetic route to copper (zinc) octa(3,5-pentoxycarbonylphenoxy)phthalocyaninates; Figure S2. SEM-images of the PEDOT–CuPc 1:4 (a), 1:6 (c) and PEDOT-ZnPc 1:4 (b), 1:6 (d) films on FTO-electrodes; Figure S3. Typical EDX spectrum of PEDOT-CuPc films on FTO-electrode.
