# Supplementary material for: Templating Effect of Water-Soluble Anionic Phthalocyaninate on the Electropolymerization of 3,4-Ethylenedioxythiophene
Source: Polymers (Basel). 2023 Apr 12;15(8):1854. doi: 10.3390/polym15081854 (PMC10143915; doi:10.3390/polym15081854)
Supplement: Supplementary file 1 [file polymers-15-01854-s001.zip › polymers-2259416-supplementary.pdf]

Supplementary materials

Templating Effect of Water-Soluble Anionic Phthalocyaninate on Electropolymerization of 3,4-Ethylenedioxythiophene

Oxana Gribkova, Varvara Kabanova, Alexey Yagodin, Aleksey Averin, Maria Teplonogova, Alexander Martynov and Alexander Nekrasov *

1. Synthesis of copper (zinc) octa(3,5-pentoxycarbonylphenoxy)phthalocyaninates

Precursor 4,5-[(3,5-bismethoxycarbonyl)phenoxy]phthalonitrile was synthesized from 4,5-dichlorophthalonitrile and dimethyl 5-hydroxyisophthalate according to the previously reported procedure [J. Med. Chem. 2005, 48, 4, 1033–1041]. Both phthalocyanines (CuPc, ZnPc) were synthesized according to the procedure described in [J. Med. Chem. 2005, 48, 4, 1033–1041] by cyclotetramerization of the precursor (Fig. S1), followed by alkaline hydrolysis of ester groups to produce well-defined complexes containing sixteen carboxylate groups. The structure of ZnPc was characterized by NMR spectroscopy and the presence of sixteen carboxylate groups can be clearly seen in the X-ray structure as reported previously [J. Med. Chem. 2005, 48, 4, 1033–1041].

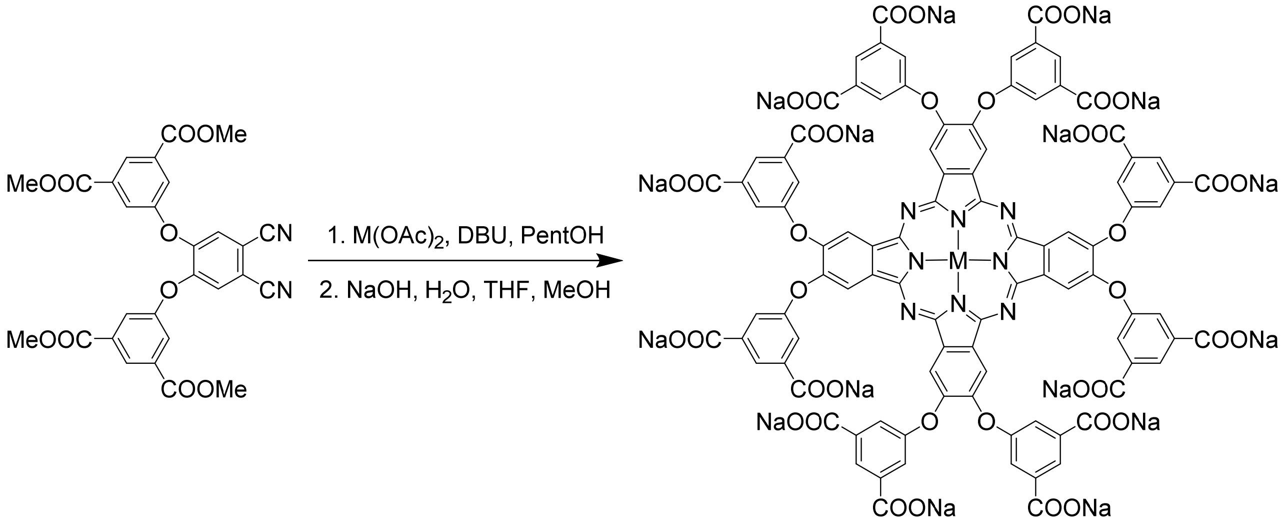

Figure S1. Synthetic route to copper (zinc) octa(3,5-pentoxycarbonylphenoxy)phthalocyaninates: M = Cu, Zn.

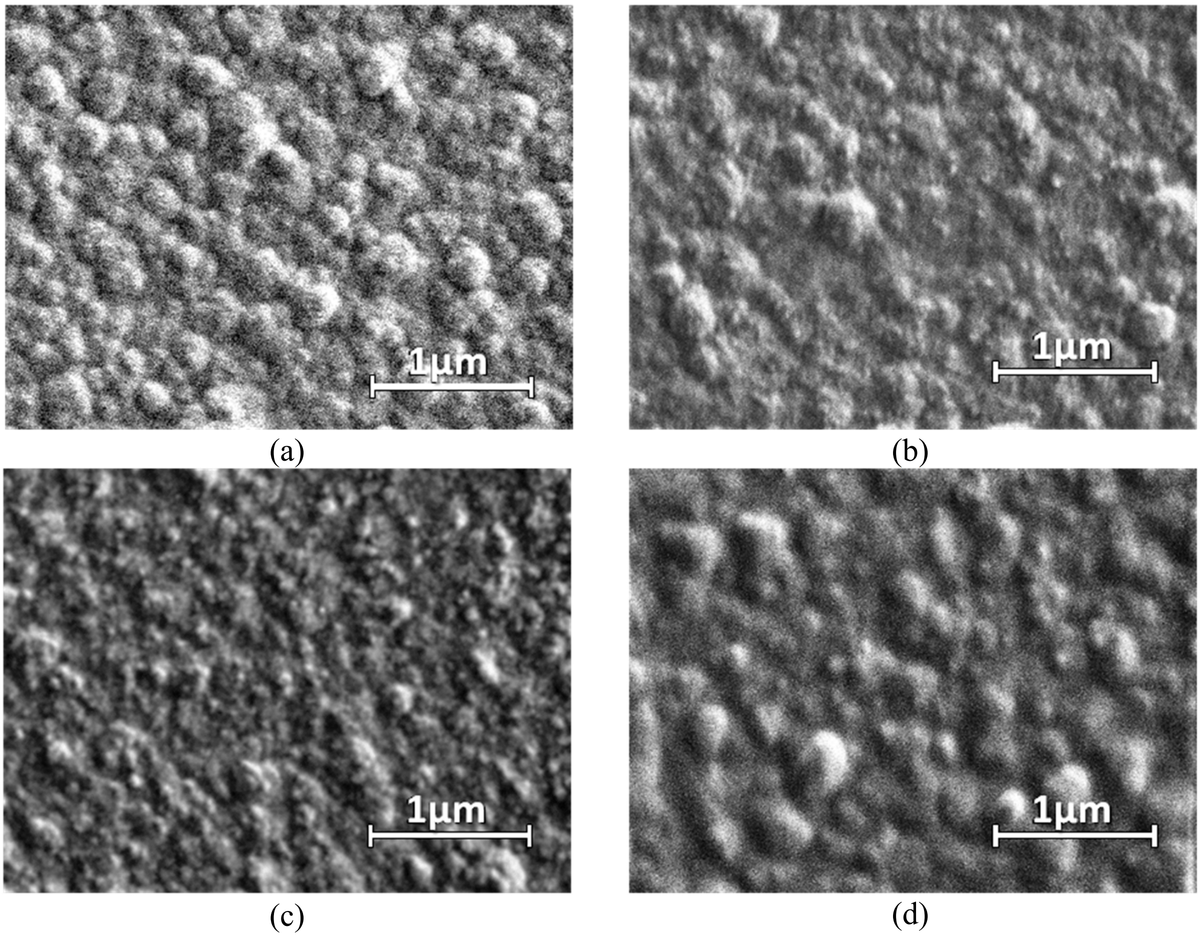

Figure S2. SEM-images of the PEDOT–CuPc 1:4 (a), 1:6 (c) and PEDOT-ZnPc 1:4 (b), 1:6 (d) films on FTO-electrodes.

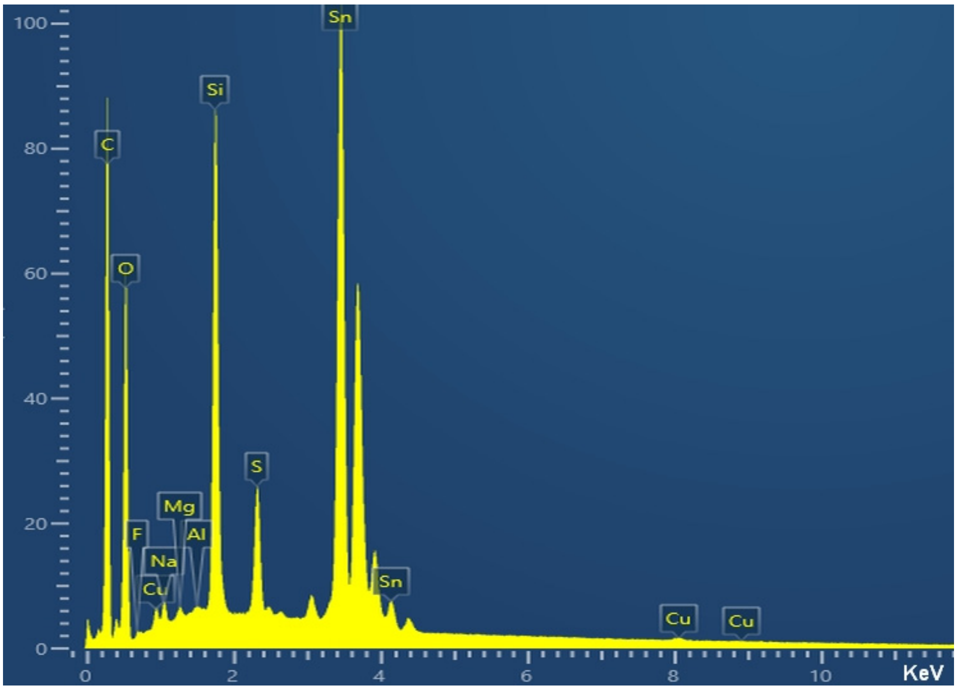

Figure S3. Typical EDX spectrum of PEDOT-CuPc films on FTO-electrode.
